# Nogo‐B promotes tumor angiogenesis and provides a potential therapeutic target in hepatocellular carcinoma

**DOI:** 10.1002/1878-0261.12358

**Published:** 2018-10-26

**Authors:** Hao Cai, Hexige Saiyin, Xing Liu, Dingding Han, Guoqing Ji, Bo Qin, Jie Zuo, Suqin Shen, Wenbo Yu, Jiaxue Wu, Yanhua Wu, Long Yu

**Affiliations:** ^1^ State Key Laboratory of Genetic Engineering Institute of Genetics School of Life Sciences Fudan University Shanghai China; ^2^ Department of Liver Surgery Liver Cancer Institute Zhongshan Hospital Key Laboratory of Carcinogenesis and Cancer Invasion Ministry of Education Fudan University Shanghai China; ^3^ Present address: Shanghai Municipal Hospital of Traditional Chinese Medicine Shanghai University of Traditional Chinese Medicine Shanghai China; ^4^ Present address: CAS Key Laboratory of Computational Biology 320 Yueyang Road Shanghai 200031 China

**Keywords:** blocking antibody, hepatocellular carcinoma, integrin, Nogo‐B, tumor angiogenesis

## Abstract

Tumor angiogenesis is one of the hallmarks of cancer as well as an attractive target for cancer therapy. Characterization of novel pathways that act in parallel with the VEGF/VEGFR axis to promote tumor angiogenesis may provide insights into novel anti‐angiogenic therapeutic targets. We found that the expression level of Nogo‐B is positively correlated with tumor vessel density in hepatocellular carcinoma (HCC). While Nogo‐B depletion inhibited tumor angiogenesis, Nogo‐B overexpression promoted tumor angiogenesis in a tumor xenograft subcutaneous model of the human HCC cell line. Mechanically, Nogo‐B regulates tumor angiogenesis based on its association with integrin α_v_β_3_ and activation of focal adhesion kinase. Moreover, Nogo‐B antibody successfully abolished the function of Nogo‐B in tumor angiogenesis *in vitro* and *in vivo*. Collectively, our results strongly suggest that Nogo‐B is an important tumor angiogenic factor and blocking Nogo‐B selectively inhibits tumor angiogenesis.

**Abbreviations**
cisPTase: cis‐prenyltransferase
CLB: cell lysis buffer
CNS: central nervous system
DAPI: 4, 6‐diamidino‐2‐phenylindole
FAK: focal adhesion kinase
HCC: hepatocellular carcinoma
HUVECs: human umbilical vein endothelial cells
MVA: mean blood vessel area fraction
MVD: mean blood vessel density
PL: polylysine
RTN4: reticulon 4
TMA: tissue microarrays
VEGFR: vascular endothelial growth factor receptor
VEGF: vascular endothelial growth factor

## 1 Introduction

Tumor angiogenesis is one of the cancer hallmarks that is shared by almost all types of human solid tumors and has become an attractive target for cancer therapy (Hanahan and Weinberg, 2011; Jayson *et al*., 2016). Vascular endothelial growth factors (VEGFs) and their cognate receptors (VEGFRs) are the key factors that are not only important for physiological angiogenesis, but also vital for tumor angiogenesis (Ferrara, 2002; Hanahan and Folkman, 1996; Millauer *et al*., 1994). Not surprisingly, the humanized monoclonal anti‐VEGF‐A antibody bevacizumab, also known as Avastin, has been approved by the FDA as a first‐line therapy for metastatic colorectal cancer (Willett *et al*., 2004). Developed later, bevacizumab is widely used to treat many other tumors, such as glioblastoma, non–small‐cell lung cancer, metastatic kidney cancer, and others. However, tumor progression eventually occurs after bevacizumab treatment in many patients with cancer (Casanovas *et al*., 2005). The limited therapeutic effect of bevacizumab has also been seen in patients with other types of cancers (Bergers and Hanahan, 2008). The clinical observations imply that angiogenic mechanisms beyond the VEGF‐A/VEGFR‐2 axis might exist in different tumor types and/or at various stages of neoplastic progression (Jayson *et al*., 2016).

Nogo‐B (also named RTN4B/RTN‐XS/Foocen‐M) belongs to the reticulon 4 (RTN4) protein families, which consists of three major splicing isoforms (Nogo‐A, Nogo‐B, and Nogo‐C) with distinct expression patterns (Cai *et al*., 2005; Yang *et al*., 2000). Nogo‐A, abundantly expressed in the central nervous system (CNS), is a key negative regulator of axonal regeneration and angiogenesis in CNS (Chen *et al*., 2000; Walchli *et al*., 2013). Nogo‐C is highly expressed in skeletal muscle and involved in cardiomyocyte apoptosis (Jia *et al*., 2016). In contrast, we previously found that Nogo‐B is widely expressed in all normal human tissues, except the liver (Cai *et al*., 2005). An essential role of Nogo‐B in regulating vascular remodeling was reported in Nogo‐A/B‐deficient mice (Acevedo *et al*., 2004). Mice deficient in Nogo‐A/B exhibit reduced arteriogenesis and angiogenesis *in vivo* due to impaired macrophage infiltration (Kondo *et al*., 2013; Yu *et al*., 2009). It has been recently demonstrated that Nogo‐B can control vascular function through suppressing endothelial sphingolipid homeostasis (Cantalupo *et al*., 2015). The function of endogenous Nogo‐B during liver disease or regeneration has also been studied using Nogo‐A/B‐deficient mice. The absence of Nogo‐B ameliorates liver fibrosis and portal hypertension after bile duct ligation (Tashiro *et al*., 2013; Zhang *et al*., 2011). Nogo‐B was upregulated and further facilitated hepatocyte proliferation and promoted liver regeneration after partial hepatectomy (Gao *et al*., 2013). Nogo‐B‐positive Kupffer cells facilitate alcoholic liver disease through regulating M1/M2 cell polarization (Park *et al*., 2017).

More recently, we have reported that the expression level of Nogo‐B was upregulated in hepatocellular carcinoma (HCC), and Nogo‐B deficiency suppressed the tumor growth and metastasis, suggesting that Nogo‐B plays an important role in HCC development (Zhu *et al*., 2017). Here, we further demonstrated that the expression level of Nogo‐B was positively correlated with tumor vessel density in HCC. We also provide evidences that Nogo‐B is a positive regulator of tumor angiogenesis *in vivo*, and anti‐Nogo‐B antibody inhibits tumor growth *in vivo* via suppressing tumor angiogenesis, suggesting that Nogo‐B is a potential therapeutic target for tumor angiogenesis.

## 2 Materials and methods

### 2.1 Tumor specimens

Surgical specimens of HCC, including tumor tissues and their adjacent nontumorous liver tissues, were collected from Zhongshan Hospital (Fudan University, Shanghai, China). Most specimens were fixed in formalin and embedded in paraffin. This work was accomplished with the approval of the Ethics Committee of School of Life Sciences of Fudan University according to the Declaration of Helsinki. Written informed consents were obtained from all patients to approve the use of their tissues for research purposes.

### 2.2 Tissue microarrays (TMA) analysis

Matched pairs of tumor samples and adjacent normal tissues from HCC, esophageal squamous cell carcinoma, gastric adenocarcinoma, renal clear cell carcinoma, rectal tubular adenocarcinoma, papillary thyroid carcinoma, and lung squamous cell carcinoma were used to construct a TMA (Shanghai Biochip Co., Ltd. Shanghai, China). In brief, sections (4 μm thickness, 1 or 2 mm diameter) were taken from individual paraffin‐embedded tissues and precisely arrayed on 3‐aminopropyltriethoxysilane–coated slides for subsequent staining with an anti‐Nogo‐B antibody.

### 2.3 Immunohistochemistry

Paraffin‐embedded specimens were cut into 5‐μm‐thick sections, deparaffinized, and rehydrated through a decreasing ethanol gradient. Endogenous peroxidase was first blocked with H_2_O_2_. After BSA blocking, slides were incubated with anti‐Nogo‐B (1 : 200 dilution; Santa Cruz, Biotechnology, Santa Cruz, CA, USA) or anti‐CD34 antibody (1 : 100 dilution; Abcam, Cambridge, UK), which was followed by incubation with biotinylated secondary antibody (1 : 100 dilution; Boster, Wuhan, China). The presence of the avidin–biotin complex was finally revealed with diaminobenzidine. Quantitative analysis of the Nogo‐B intensity, CD34‐positive blood vessel density, and blood vessel area was performed using imagej software.

### 2.4 Cell lines, cell culture, and cell transfection

SMMC‐7721 was purchased from the Shanghai Institute for Biological Sciences, Chinese Academy of Sciences (Shanghai, China). SK‐Hep1, CHO, and HEK293T cell lines were purchased from ATCC (Manassas, VA, USA). All cells are maintained in Dulbecco's modified essential medium supplemented with 10% fetal bovine serum. G418 (800 μg·mL^−1^; Invitrogen, Waltham, MA, USA) was used to maintain stable SMMC‐7721 lines. Primary human umbilical vein endothelial cells (HUVECs) were purchased from ScienCell (Carlsbad, CA, USA) and maintained in M200 medium supplemented with 2% LSGS (Cascade Biologics, Portland, OR, USA), penicillin (50 U·mL^−1^), and streptomycin (50 mg·mL^−1^). Cells in passages 3–8 were used in the experiments. The above cells were cultured at 37 °C in a humidified 5% CO_2_ atmosphere. Cells at 80% confluency were transfected with the indicated plasmids or small interference RNA (siRNA) using Lipo2000 (Invitrogen) according to the manufacturer's protocol.

### 2.5 Small interference RNA screen and lentivirus infection

Nogo‐B siRNA1 (S1; forward seq: 5ʹ‐UUGGCACAGAUAGAUCAUUAU‐3ʹ), siRNA2 (S2; forward seq: 5ʹ‐UUCAGAAUCUAUGGACUGAAU‐3ʹ), and nonsilencing control (NS; forward seq: 5ʹ‐UUCUCCGAACGUGUCACGU‐3ʹ) were designed and constructed into lentiviral shRNA plasmid at Shanghai Genechem Co., Ltd. (Shanghai, China). The corresponding lentiviral particles were packaged and designated as LRS1, LRS2, and LNS, respectively. SMMC‐7721 cells cultured in 96‐well plates were infected with lentivirus at a multiplicity of infection of 10. The silencing effect was examined by immunoblot 72 h after infection.

### 2.6 Human xenograft subcutaneous tumor assay

This work was accomplished with the approval of the Ethics Committee of School of Life Sciences, Fudan University. Animal experiments were carried out in accordance with the guidelines for the Care and Use of Laboratory Animals of Shanghai Municipality, PR China. The protocol was approved by the Science and Technology Commission of Shanghai Municipality (Permit Number: SYXK 2015‐0006).

Six‐week‐old female athymic nude mice were obtained from Shanghai Laboratory Animal Co., Ltd. (SLAC, Shanghai, China), and maintained on standard laboratory chow under a 12 h : 12 h light–dark schedule with free access to food and water. Cultured cells were harvested and washed with the culture medium without serum and resuspended in sterile 1× PBS before tumor implantation. Three to five million viable cells in 200 μL were subcutaneously injected into the right flanks of mice. Six to eight animals were used in each group. The tumor size was measured with a caliper, and the mice were weighed every 3 days. The tumor volume was calculated using the formula of length × width^2^ × 0.5. Four weeks after injection, animals were sacrificed by neck dislocation to minimize suffering, and the tumors were collected and weighed. Fresh tumor samples were fixed in freshly prepared 4% PFA overnight before further analysis.

### 2.7 Immunofluorescence staining

Xenografted tumor samples were fixed in freshly prepared 4% PFA overnight. Frozen samples were cut into 4‐μm slides and hydrated. Immunofluorescence staining was performed as previously described. After blocking with 5% BSA and 10% donkey serum, slides were incubated with anti‐CD31/PECAM antibody (1 : 20; BD Biosciences, San Jose, CA, USA) or anti‐phosphorylated focal adhesion kinase (FAK) antibody (1 : 20; Abcam) overnight at 4 °C, which was followed by Cy3‐conjugated secondary antibody (1 : 100; Zymed Laboratories, South San Francisco, CA, USA). The slides were counterstained with DAPI (4, 6‐diamidino‐2‐phenylindole; Sigma, St. Louis, MO, USA). All images were acquired using a LEICA DC 500 camera on a microscope equipped with LEICA DMRA2 fluorescent optics (LEICA, Buffalo Grove, IL, USA). Quantitative analysis of the CD31‐positive blood vessel density and phosphorylated FAK‐positive area was performed using imagej software.

### 2.8 Cell adhesion assay

Recombinant Nogo‐B was expressed in the Pichia expression system (Invitrogen) according to K. Sreekrishna's protocol. His‐tagged Nogo‐B in cell lysate was further purified with a Ni‐NTA Spin Kit (QIAGEN, Hilden, Germany) according to the manufacturer's instructions. HUVECs were used in the cell adhesion assay. A 96‐well plate was coated with proteins diluted in phosphate‐buffered saline (PBS) at 4 °C overnight and then blocked with BSA. Cells were harvested with EDTA/trypsin and resuspended in serum‐free medium at 2 × 10^5^ cells·mL^−1^. Then, 100 μL of the cell suspension was plated in each well. After 1‐h incubation at 37 °C, cells were extensively washed three times with warmed PBS. Adherent cells were quantified using the MTS assay (Promega, Fitchburg, WI, USA), and the absorbance was measured with a microtiter reader (Bio‐Rad, Hercules, CA, USA) at 450 nm. In the cell adhesion blockade assay, resuspended HUVECs were incubated with indicated antibodies or peptides before they were replated into wells.

### 2.9 FAK phosphorylation assay

Cells adherent on dishes in the spreading assay were lysed on ice with cell lysis buffer (CLB; Cell Signaling, Danvers, MA, USA) supplemented with protease inhibitor cocktail (Cell Signaling), PMSF, and sodium orthovanadate. The total protein levels were determined, and the same amounts were used in immunoprecipitation. Then, 1 μg of anti‐FAK antibody was added into cell lysate and incubated for 4 h at 4 °C, which was followed by incubation with Protein A/G agarose beads for another 45 min at 4 °C. The immunocomplexes were washed with the CLB containing additional PMSF and sodium orthovanadate four times before western blot analysis. For immunoblotting, blots were incubated with anti‐FAK antibody (1 : 500; Merck, Darmstadt, Germany) and anti‐tyrosine/phosphorylation antibody (1 : 500; Merck), respectively.

### 2.10 Tube formation assay

Matrigel (BD Biosciences) was used to coat the wells of 96‐well plates (50 μL per well) and was allowed to polymerize at 37 °C for 1 h. HUVECs were harvested and suspended in serum‐free medium at 2.4 × 10^5^ cells·mL^−1^. Cells (50 μL) were then mixed with either Nogo‐B, VEGF (Peprotech, Rocky Hill, CT, USA) as a positive control, or PBS as a negative control diluted in serum‐free medium (50 μL) before they were seeded onto the Matrigel surface. After 6–10 h, HUVECs were photographed and the extent of tube formation was analyzed using imagej software.

### 2.11 *In vivo* Matrigel angiogenesis assay

Recombinant proteins were incorporated in Matrigel (BD Biosciences) with heparin sulfate (40 U; Sigma). Six‐week‐old female C57BL/6 mice (SLAC) were subcutaneously injected with 0.5 mL of Matrigel in combination with Nogo‐B, VEGF as a positive control, or PBS as a negative control. Mice were sacrificed after 7–10 days. The Matrigel plugs were dissected and photographed. Then, the specimens were fixed in freshly prepared 4% PFA overnight and further subjected to immunohistochemical staining using anti‐CD31/PECAM antibody (1 : 20; BD Biosciences). Quantitative analysis of the CD31‐positive blood vessel area was performed using imagej software.

### 2.12 Antibody generation and purification

A peptide corresponding to amino acids 44–58 of human Nogo‐B was synthesized at China Peptides Corporation (Shanghai, China) and used to immunize mice to produce monoclonal anti‐Nogo‐B antibody through the hybridoma technique at AbMax Biotechnology Co., Ltd. (Beijing, China). In brief, 4‐ to 8‐week‐old Balb/c mice were immunized with 500 μg of synthesized Nogo‐B peptide. Serum titers were assessed with the ELISA before the spleen cells were fused with SP2/0 cells. Hybridomas were selected, and their culture supernatants were screened for the presence of antibody through ELISA. Positive hybridomas were further cloned using the limiting dilution technique. Monoclonal antibody in the hybridoma clone culture supernatant was purified using protein A Sepharose columns (GE Healthcare, Little Chalfont, UK) according to the manufacturer's instructions.

### 2.13 Statistical analysis

Group‐level differences were evaluated through a two‐tailed Student's *t*‐test. The relationship between the MVD/MVA and Nogo‐B intensity was analyzed using a two‐tailed Pearson correlation. *P* < 0.05 (*) was considered significant, and *P* < 0.01 (**) was considered highly significant.

## 3 Results

### 3.1 Nogo‐B expression correlates with high blood vessel density in human HCC tissues

**Figure 1:**
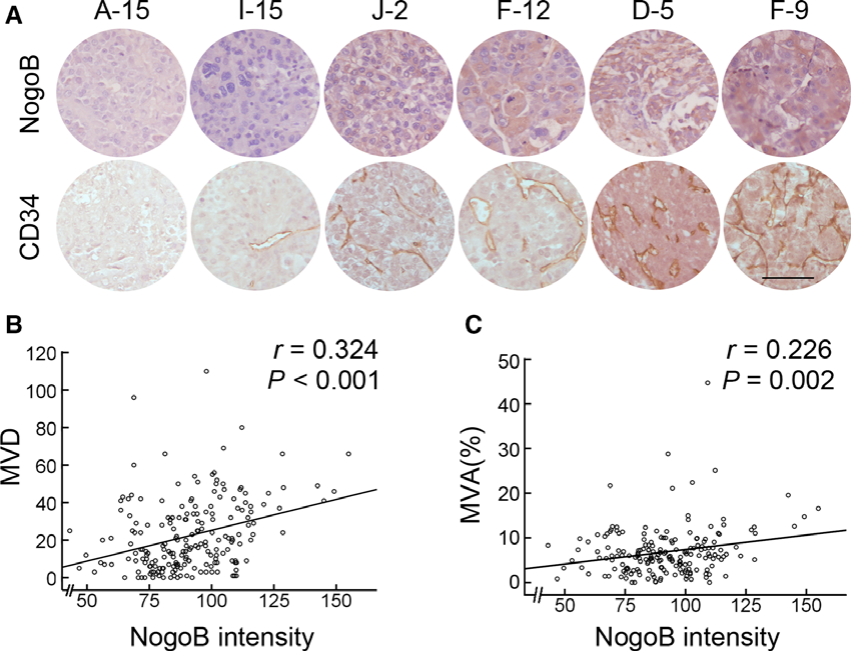
Nogo‐B expression correlates with tumor angiogenesis in human HCC. (A) Representative photographs of immunohistochemistry analysis of Nogo‐B and CD34 expression in a human HCC TMA. Scale bar, 100 μm. (B, C) The relationship between the CD34‐positive blood vessel density (MVD) (B) and blood vessel area fraction (MVA) (C) with Nogo‐B expression in 211 HCC cases. Statistical significance was evaluated by Spearman's correlation.

More recently, we have found that the expression level of Nogo‐B was upregulated in HCC specimens (Zhu *et al*., 2017). As Nogo‐B is a regulator of angiogenesis in mice (Acevedo *et al*., 2004), Nogo‐B may play an important role in tumor angiogenesis. To test this hypothesis, we firstly examined the expression levels of Nogo‐B and CD34, a marker of vascular, in two TMA containing 211 cases of HCC specimens by immunohistochemical staining using anti‐Nogo‐B and anti‐CD34 antibodies, respectively (Fig. S1). Representative photographs of HCC specimens with staining for Nogo‐B and CD34 are shown in Fig. 1A. Tumor blood vessel formation was obviously increased in HCC tissues with higher expression of Nogo‐B compared with tumors with low Nogo‐B expression. Moreover, statistical analysis also revealed that Nogo‐B expression was positively correlated with both blood vessel density (MVD) and blood vessel area fraction (MVA) in HCC tissues (Pearson correlation coefficient; *P* < 0.01) (Fig. 1B,C). These results suggested that Nogo‐B may play an important role in tumor angiogenesis.

### 3.2 Overexpression of Nogo‐B in HCC cells promotes tumor angiogenesis *in vivo*

**Figure 2:**
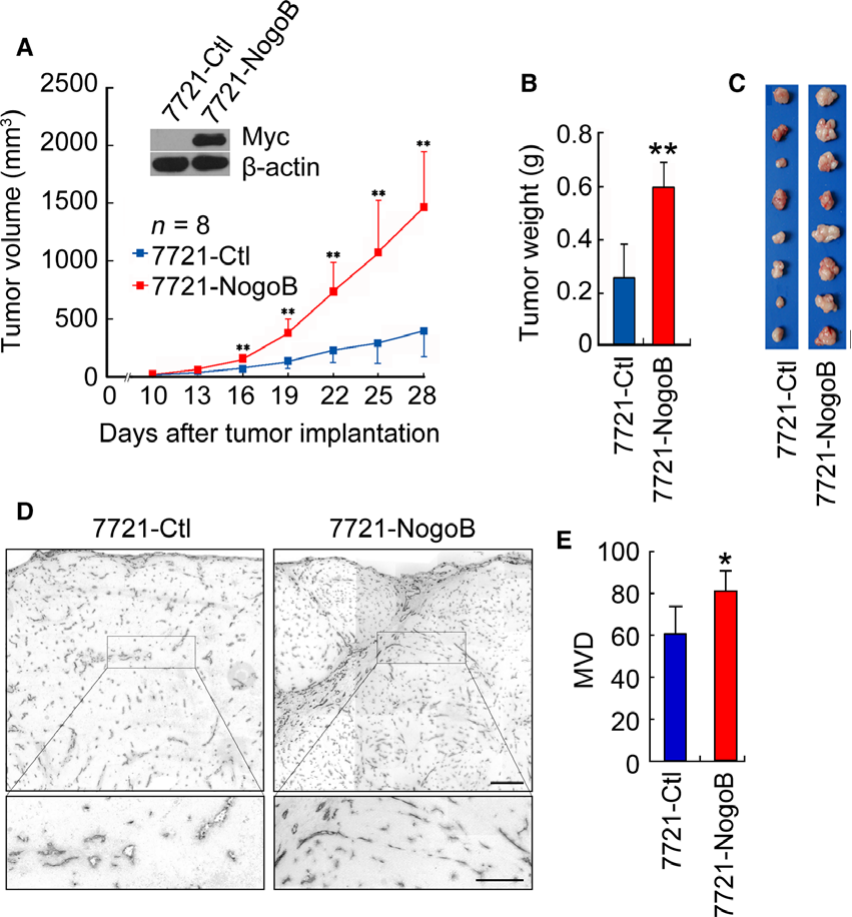
Nogo‐B overexpression promotes tumor growth and angiogenesis. (A) Measurement of tumor size in nude mice engrafted with SMMC‐7721 cells stably expressing Nogo‐B (7721‐Nogo‐B) or control cells (7721‐Ctl). Error bars represent the SD (*n* = 8, **P < 0.01). Western blot analysis of exogenous Nogo‐B expression in two cell sublines is shown in the upper panel. β‐actin serves as a loading control. (B) Measurement of the xenograft tumor weight. The error bar represents the SD (*n* = 8, **P < 0.01). (C) Photographs of xenograft tumors dissected from the mice as indicated. Scale bars, 10 mm. (D) Immunohistochemistry with a CD31/PECAM‐1 antibody of tumor sections. Small rectangles in the upper panel (scale bars, 200 μm) are shown with higher magnification in the bottom panel (scale bars, 50 μm). (E) Quantification of MVD in tumor sections randomly selected by morphometry. The error bar represents the SD (*n* = 5, *P < 0.05).

To investigate the function of Nogo‐B in tumor angiogenesis, we generated stably expressed Myc‐tagged Nogo‐B SMMC‐7721 cells. Nogo‐B‐overexpressed cells display dramatically increased tumor growth, tumor size, and tumor weight *in vivo* after subcutaneous injection into nude mice compared with control cells (Fig. 2A–C). Next, we examined the blood vessel density in the tumor histological sections derived from control cells or Nogo‐B‐overexpressed cells by immunohistochemical staining using anti‐CD31 antibody. As shown in Fig. 2D, tumor sections derived from Nogo‐B‐overexpressing cells exhibited stronger immunoreactivity of anti‐CD31 antibody than those from the control cells. Quantitatively, the mean CD31‐positive MVD in Nogo‐B‐derived tumor sections was increased by ~ 30% (*P* < 0.01, *t*‐test) compared with control tumor sections (Fig. 2E). These results further indicate that Nogo‐B might be involved in regulating tumor angiogenesis *in vivo*.

### 3.3 Knockdown of Nogo‐B in HCC cells suppresses tumor angiogenesis *in vivo*

**Figure 3:**
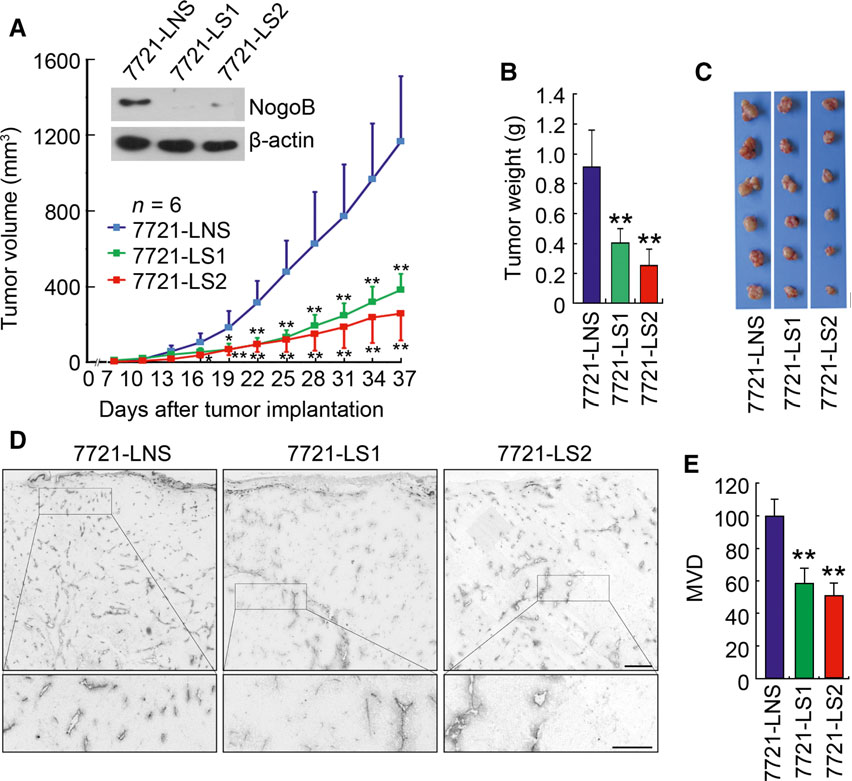
Knockdown of endogenous Nogo‐B suppresses tumor growth and angiogenesis. (A) Measurement of the tumor size in nude mice engrafted with SMMC‐7721 cells infected with lentivirus expressing shNogo‐B‐1 (7721‐LS1), shNogo‐B‐2 (7721‐LS2), or nonsilencing shRNA control (7721‐LNS). Error bars represent the SD (*n* = 6; *P < 0.05; **P < 0.01). Western blot analysis of endogenous Nogo‐B expression in three cell sublines is shown in the upper panel. β‐actin serves as a loading control. (B) Measurement of the xenograft tumor weight. The error bar represents the SD (*n* = 6, **P < 0.01). (C) Photographs of tumors dissected from nude mice engrafted with 7721‐LRS1, 7721‐LRS2, and control cells (7721‐LNS). Scale bars, 10 mm. (D) Immunohistochemical staining with a CD31/PECAM‐1 antibody of tumor sections. Small rectangles in the upper panel (scale bars, 200 μm) are shown with higher magnification in the bottom panel (scale bars, 50 μm). (E) Quantification of the MVD in tumor sections randomly selected by morphometry. The error bar represents the SD (*n* = 5, *P < 0.05).

Next, we examined the role of endogenous Nogo‐B in tumor angiogenesis. To this end, a lentivirus‐based RNA interference approach was used to specifically silence endogenous Nogo‐B in SMMC‐7721 cells. Two siRNA species, siRNA1 targeting the coding region and siRNA2 targeting the 3ʹ‐UTR of Nogo‐B mRNA, were designed, and two corresponding lentivirus‐based expression constructs, LRS1 and LRS2, were subsequently generated. A nonspecific control siRNA and its lentivirus expression vector (LNS) were also designed. Consistent with the results of stable expression of Nogo‐B, knocking down Nogo‐B exhibited suppressive effect in HCC cell growth *in vivo* (Fig. 3A–C). At the time of animal euthanasia, the 7721‐LS1 and 7721‐LS2 tumors showed a remarkable decrease in the tumor size of 67% and 78% (*P* < 0.01, *t*‐test), respectively, compared with control tumors. Consistent with these findings, the tumor weights of the 7721‐LS1 and 7721‐LS2 groups were decreased by 55% and 73% (*P* < 0.01, *t*‐test), respectively.

Again, immunohistochemistry of CD31/PECAM was performed to examine whether endogenous Nogo‐B knockdown affects tumor angiogenesis. As shown in Fig. 3D, histological sections from 7721‐LRS1‐ and 7721‐LRS2‐derived tumors had much weaker immunoreactivity with the anti‐CD31/PECAM antibody. Quantitatively, the mean CD31‐positive vessel densities in tumors derived from Nogo‐B‐knockdown cells were significantly decreased by 41% and 49% (*P* < 0.01, *t*‐test), respectively (Fig. 3E). These results provide further evidence that Nogo‐B probably plays an important role in HCC tumor growth and angiogenesis.

### 3.4 Recombinant Nogo‐B regulates cell adhesion in an integrin α_v_β_3_‐dependent manner

**Figure 4:**
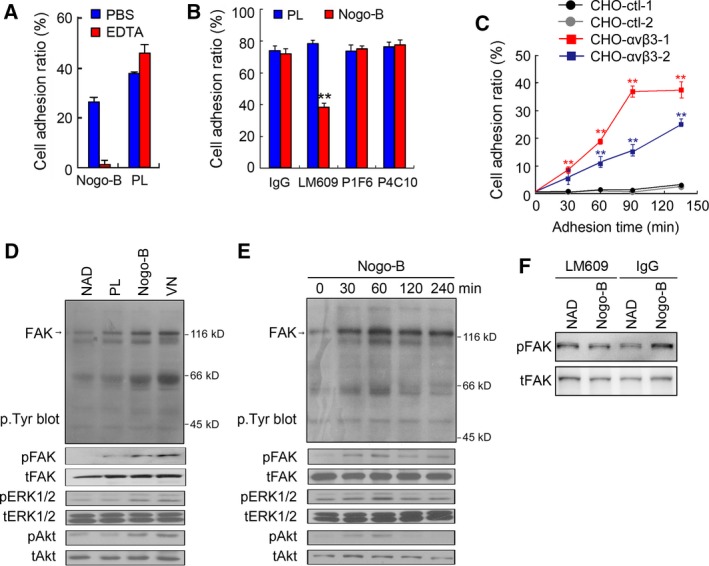
Recombinant Nogo‐B promotes cell adhesion through interacting with integrin α_v_β_3_. (A) Effects of EDTA on HUVEC adhesion. Cells were incubated with EDTA or PBS before they were replated on Nogo‐B‐coated surfaces. (B) The cell adhesion blockade assay to investigate the effects of integrin‐neutralizing antibodies on Nogo‐B‐induced HUVEC adhesion. Cells were incubated with neutralizing anti‐integrin antibodies as indicated before they were replated on Nogo‐B‐coated surfaces. LM609, anti‐integrin α_v_β_3_ antibody; P1F6, anti‐integrin α_v_β_5_ antibody; and P4C10, anti‐integrin β_1_ antibody. Polylysine was used as an integrin‐independent control. The error bar represents the SD (*n* = 3, **P < 0.01). (C) Effect of Nogo‐B expression on CHO cell adhesion. Two CHO control cell lines (CHO‐ctl‐1, CHO‐ctl‐2) and two CHO stable cell lines expressing integrin α_v_β_3_ (CHO‐α_v_β_3_‐1, CHO‐α_v_β_3_‐2) were replated on the Nogo‐B‐coated surface, and cell adherence was determined at different time points. The error bar represents the SD (*n* = 3, **P < 0.01). (D) FAK phosphorylation assay. The levels of indicated phosphorylated and total protein were detected in whole cell lysate (WCL) from cells adhered to PL, Nogo‐B, or vitronectin (VN)‐coated surfaces or cells in suspension (NAD). (E) Time course studies of different protein phosphorylation for cells grown on Nogo‐B‐coated surfaces. (F) FAK phosphorylation analysis after HUVEC was blocked by integrin α_v_β_3_ neutralizing antibody.

The recombinant Nogo‐B was expressed and purified to analyze the function in angiogenesis. Integrins are a large family of cell‐surface receptors that play critical roles in mediating several angiogenic steps, including cell adhesion and cell migration. Integrin‐mediated cell adhesion is sensitive to Ca^2+^ sequestration and can be blocked by EDTA treatment (Hynes, 2002). It is found that EDTA treatment can dramatically inhibit the function of Nogo‐B on the cell adhesion of HUVECs, while it did not inhibit adherence to the polylysine (PL)‐coated surfaces (Fig. 4A). Moreover, the antibody specific to integrin α_v_β_3_ (LM609), but not integrin α_v_β_5_ (P1F6) or the β_1_ family (P4C10), significantly inhibited the adherence of HUVECs to Nogo‐B‐coated plates (Fig. 4B). Next, the CHO cell line, which lacks endogenous integrin β_3_ expression, was used to determine whether integrin α_v_β_3_ is sufficient to mediate Nogo‐B‐regulated cell adhesion. Stable CHO cell sublines expressing myc‐tagged integrin β_3_ were generated (Fig. S2a), which could form an α_v_β_3_ heterodimer with an endogenous α_v_ subunit on the cell membrane, as confirmed by flow cytometric analysis using the anti‐integrin α_v_β_3_ antibody (Fig. S2b). As expected, only β_3_‐overexpressing CHO clones, but not control CHO cells, could adhere to Nogo‐B‐coated surfaces (Fig. 4C).

Upon ligand occupancy, integrins transduce outside signals into cells to initiate cytoskeletal re‐organization via recruiting protein kinases, scaffolding molecules, and cytoskeleton components to organize the focal adhesion complex (Miranti and Brugge, 2002). Therefore, we sought to determine whether Nogo‐B regulates the actin cytoskeletal structure and focal contact remodeling by immunoblot with specific antibodies. As shown in Fig. 4D, attachment of cells to Nogo‐B, but not negative control PL, increased the intensity of several proteins reacted with the anti‐p.Tyr antibody, which was similar to the attachment to vitronectin, the known ligand for integrin α_v_β_3_. Compared with control treatment, tyrosine phosphorylation of FAK and phosphorylation of its downstream factors, ERK1/2 and Akt, are also induced by Nogo‐B and vitronectin treatment. Furthermore, in a time‐gradient period, FAK, ERK1/2, and Akt phosphorylation peaked at 60 min after cells were replated on Nogo‐B‐coated plates (Fig. 4E). Moreover, the phosphorylation of FAK induced by Nogo‐B was blocked when HUVEC was treated with integrin α_v_β_3_‐neutralizing antibody (Fig. 4F). These results together suggest that the interaction between Nogo‐B and integrin α_v_β_3_ could activate the FAK‐initiated signaling pathway, strengthening the cell adhesion and migration properties.

### 3.5 Nogo‐B antibody suppressed tumor angiogenesis *in vitro* and *in vivo*

**Figure 5:**
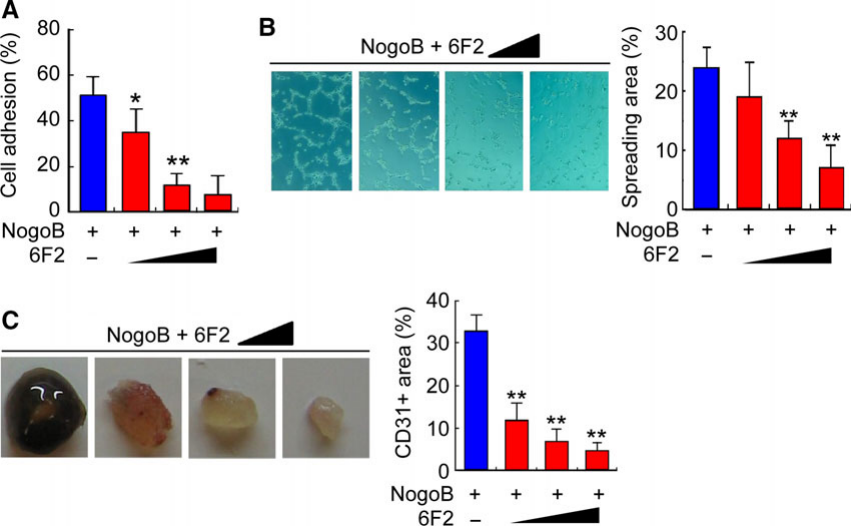
Anti‐Nogo‐B antibody 6F2 suppresses angiogenesis *in vitro*. (A) The cell adhesion blockage assay in which HUVECs were incubated with mAb 6F2 before they were replated on Nogo‐B‐coated surfaces. The error bar represents the SD (*n* = 3, **P < 0.01). (B) The tube formation blockage assay in which HUVECs were incubated with both recombinant Nogo‐B and mAb 6F2 before they were replated on Matrigel‐coated surfaces. Quantitative analysis was performed by counting the tube area fraction. The error bar represents the SD (*n* = 3, **P < 0.01). (C) Matrigel inhibition assay in which BALB/c mice were injected with Matrigel containing both recombinant Nogo‐B and mAb 6F2. Quantitative analysis was performed by counting the CD31‐positive blood vessel area fraction. The error bar represents the SD (*n* = 3, **P < 0.01).

How tumor vessels are formed and remodeled is of great interest for developing effective anti‐angiogenic drugs to restrict tumor growth (Jayson *et al*., 2016). After we revealed the potential function of Nogo‐B in regulating tumor angiogenesis, we sought to determine whether blocking Nogo‐B can be a novel therapeutic strategy for HCC. To this end, a monoclonal antibody against Nogo‐B, designated as 6F2, was generated by the hybridoma technique. The 6F2 epitope resides within the N‐terminal domain (44–58 aa) of Nogo‐B. The effects of 6F2 on Nogo‐B‐mediated cellular function were first examined with the cell adhesion assay, tube formation assay, and Matrigel plug assay. As shown in Fig. 5A–C, 6F2 treatment attenuated Nogo‐B‐induced cell adhesion, the tube spreading area, and the CD31‐positive blood vessel area in Matrigel plug in a dose‐dependent manner, respectively.

**Figure 6:**
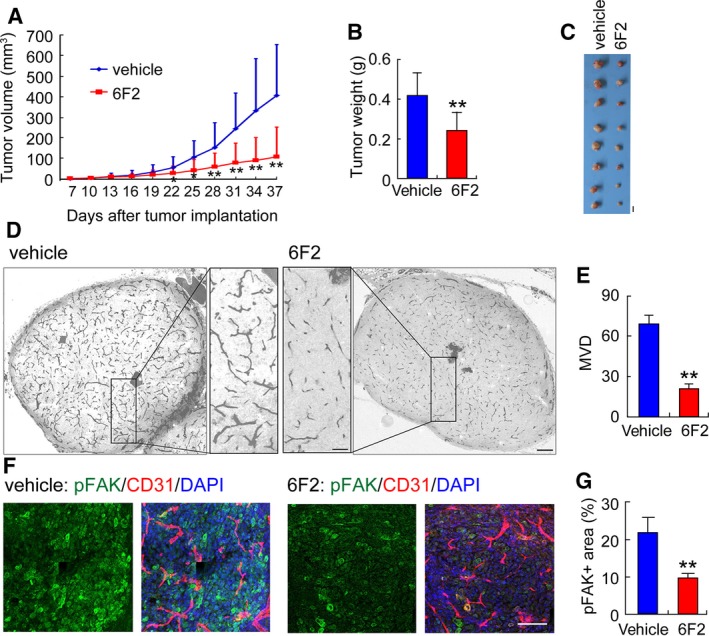
Anti‐Nogo‐B antibody 6F2 suppresses angiogenesis *in vivo*. (A, B) Measurement of the tumor size (A) and tumor weight (B) in nude mice engrafted with SMMC‐7721 wild‐type cells given 6F2 (20 mg per kg of body weight) or vehicle by i.p. injection. Error bars represent the SD (*n* = 8; *P < 0.05; **P < 0.01). (C) Photographs of tumors dissected from nude mice engrafted with SMMC‐7721 wild‐type cells that received 6F2 or vehicle by i.p. injection. Scale bars, 10 mm. (D) Immunohistochemical staining with a CD31/PECAM‐1 antibody of tumor sections. Small rectangles (scale bars, 200 μm) are shown at higher magnification in the inner panel (scale bars, 50 μm). (E) Quantification of the MVD in tumor sections randomly selected by morphometry. The error bar represents the SD (*n* = 5, *P < 0.05). (F) Representative photographs of tumor sections co‐immunostained with pFAK, CD31, and nuclear stain. Scale bars, 100 μm. (G) Quantification of the randomly pFAK‐positive area fraction selected with morphometry. The error bar represents the SD (*n* = 5, *P < 0.05).

Next, the effect of 6F2 in tumor and tumor angiogenesis was examined *in vivo*. SMMC‐7721 wild‐type cells were subcutaneously injected into nude mice, and animals were then received 6F2 (20 mg per kg of body weight) or vehicle by i.p. injection. As shown in Fig. 6A–C, both the tumor size (73%) and tumor weight (43%) (*P* < 0.01, *t*‐test) were significantly decreased in 6F2‐treated mice compared with vehicle treatment. Anti‐CD31 immunostaining further revealed that 6F2 attenuated the blood vessel formation in xenograft tumors, leading to a remarkable decrease in the tumor blood vessel density (Fig. 6D,E). More specifically, the anti‐phosphorylated FAK immunosignal area was significantly suppressed by 6F2 treatment (Fig. 6F,G), indicating an inhibitory effect of 6F2 in Nogo‐B‐induced signaling transduction. These results demonstrated that Nogo‐B antibody can attenuate tumor angiogenesis *in vitro* and *in vivo*, suggesting that it might be a potential therapeutic drug for HCC.

## 4 Discussion

Most studies on the Nogo family have centered on their roles in axonal regeneration until the function of Nogo‐B in regulating endothelial cell activities and vascular function was reported in mice (Acevedo *et al*., 2004). The role of Nogo‐B in human cardiovascular disease has been further documented (Bullard *et al*., 2008). On the other hand, the function of Nogo‐B in cancer cells has been studied for at least a decade, but inconsistent results have been reported with respect to the pro‐apoptotic function of Nogo‐B and its expression pattern in cancer cells (Li *et al*., 2001; Oertle *et al*., 2003). In the beginning of our study, we investigated the expression of Nogo‐B in different cancers, and a consistent upregulated expression pattern of Nogo‐B was observed in all examined cancer types (Fig. S3), suggesting that Nogo‐B may play general and critical functions in tumor development. Given that Nogo‐B is only expressed in the sinusoidal endothelial cells but not in the hepatocytes of normal liver tissue (Fig. S4), we selected HCC cells as our working model for further functional study. We demonstrate that overexpression of Nogo‐B in HCC cells or recombinant Nogo‐B significantly promotes tumor angiogenesis, while inhibition of Nogo‐B via shRNA or neutralizing antibody impedes angiogenesis *in vitro* and *in vivo*. Our finding suggests Nogo‐B as a potential target of anti‐angiogenesis tumor therapy.

After revealing that Nogo‐B functions in vascular remodeling and angiogenesis, the functional receptor of Nogo‐B has been screened individually in several laboratories. NgBR has been characterized as a functional receptor on the endothelial cell surface that is necessary for Nogo‐B stimulated chemotaxis and angiogenesis (Miao *et al*., 2006). It is now known that NgBR is a subunit of cis‐prenyltransferase (cisPTase), which is required for dolichol monophosphate biosynthesis and protein N‐glycosylation (Harrison *et al*., 2011). Genetic knockdown of NgBR in zebrafish resulted in an intersomitic vessel defect during embryonic development, and endothelial‐specific knockout of NgBR in mice resulted in vascular development defects and embryonic lethality (Park *et al*., 2016; Rana *et al*., 2016; Zhao *et al*., 2010). However, *in vivo* studies also revealed that the NgBR functions in mouse endothelial cells during embryogenesis are Nogo‐B independent, which strongly suggests that Nogo‐B and NgBR are at least partially functionally different. Similar results have been found in the NgBR liver‐specific knockout mice. NgBR was demonstrated to be a specific negative regulator for hepatic lipogenesis, which is apparently inconsistent with the positive regulatory role of Nogo‐B during liver fibrosis/cirrhosis (Hu *et al*., 2016).

Some preliminary studies of NgBR expression in tumor cells have also been performed recently, but the results differ for different tumor types. For instance, NgBR was upregulated in breast cancer compared with normal breast tissue and the expression of NgBR promoted the chemoresistance of HCC cells (Dong *et al*., 2016; Wang *et al*., 2013). In contrast, association studies demonstrated that the expression of NgBR was negatively correlated with the malignancy grade in invasive ductal breast carcinoma, non–small‐cell lung carcinomas, and malignant melanoma (Calik *et al*., 2016; Pula *et al*., 2014a,b). Although these results suggest that NgBR might be involved in tumor development, whether NgBR acts through binding Nogo‐B in tumor cells remains to be investigated.

It is noteworthy that some other novel receptors of Nogo‐B have recently been reported, including PirB, GRAMD4, and SPT, which brings an additional degree of complexity to the molecular mechanism of Nogo‐B (Cantalupo *et al*., 2015; Kimura *et al*., 2015; Kondo *et al*., 2013). For instance, Nogo‐B suppresses the *de novo* pathway of sphingolipid biosynthesis in endothelial cells through interacting with SPT (the rate‐limiting enzyme of the pathway), but this function is independent on NgBR (Cantalupo *et al*., 2015). In our study, we demonstrated that Nogo‐B associated with integrin α_v_β_3_, raising a hypothesis that integrin α_v_β_3_ on the surface of endothelial cells is another novel receptor of Nogo‐B during tumor angiogenesis. We also provided evidence that Nogo‐B promoted HUVEC adhesion and activated the FAK‐initiated signaling pathway in an integrin α_v_β_3_‐dependent manner. Integrin α_v_β_3_ was initially believed to be required for pathological angiogenesis (Gasparini *et al*., 1998; Natali *et al*., 1997). Previous studies have reported the diametric roles of integrin α_v_β_3_ during tumor angiogenesis, which are due to its promiscuous interactions with different ligands (Hynes, 2002). Our results indicate that Nogo‐B is probably a new pro‐angiogenic ligand of integrin α_v_β_3_. Consistent with our finding, Hu *et al*. found that the N‐terminal domain of Nogo‐A inhibits cell adhesion and axonal outgrowth through several integrins, including α_v_β_3_ (Hu and Strittmatter, 2008). ER‐resident Nogo‐B protein can not only localize to the plasma membrane but also secrete into extracellular space (Acevedo *et al*., 2004; Ozkaramanli Gur *et al*., 2018). Nogo‐B in HCC cells exhibited a consistent distribution in our work, that it can be detected both on HCC cell surface and in the cell culture medium (data not shown). So we hypothesized that Nogo‐B might regulate tumor angiogenesis through directly binding to integrins on endothelial cells’ surface and/or secreting into the extracellular space.

## 5 Conclusions

The first clinically used angiogenesis inhibitor, bevacizumab, is widely applied to treat several types of cancers (Ferrara *et al*., 2004). However, the redundancy of angiogenic factors during tumor angiogenesis contributes to resistance to anti‐VEGF therapy (Bergers and Hanahan, 2008; Carmeliet and Jain, 2011; Jayson *et al*., 2016). It has been suggested that VEGF blockade aggravates hypoxia, which then drives the expression of other angiogenic proteins, such as fibroblast growth factor, angiopoietin‐2, interleukin‐8, among others (Casanovas *et al*., 2005; Huang *et al*., 2010; Rigamonti *et al*., 2014). These factors further contribute to the revascularization and regrowth of tumors. It will be fascinating in the near future to determine the outcome of combining novel anti‐angiogenic agents with traditional anti‐VEGF drugs (Smith *et al*., 2014). Our present study defines Nogo‐B/integrin as a previously uncharacterized pathway that may function in parallel with the VEGF/VEGFR axis in promoting tumor angiogenesis. The existence of the alternative Nogo‐B/integrin pathway might provide another plausible explanation for the recurrence of many types of tumors after anti‐VEGF therapy as well as represents an attractive target for the development of new effective therapeutics against tumor angiogenesis.

## Author contributions

LY and JW conceived the study, designed the experimental strategy, and participated in manuscript preparation. YW carried out *in vitro* angiogenic assays and mechanism exploration, analyzed all the data, and wrote the manuscript. HC prepared the blocking antibody, and performed the blocking assay and the xenograft tumor assay. HS performed IHC and IF, and was involved in association study and data analysis. XL screened siRNA fragment, established all stable lines, and analyzed cell activity. DH performed IF and the xenograft tumor assay. GJ was involved in experimental design, xenograft tumor assay and data analysis. BQ produced recombinant protein. JZ was involved in *in vitro* angiogenic assays. SS and WY were involved in data analysis.

## Supporting information


**Fig. S1.** Photographs of two TMAs.
**Fig. S2.** Establishment of CHO clones stably expressing integrin α_v_β_3_.
**Fig. S3.** Nogo‐B is remarkably upregulated in different cancers.
**Fig. S4.** Representative photographs of immunohistochemical Nogo‐B staining in normal liver tissue and HCC.
