# Supplementary material for: Nogo‐B promotes tumor angiogenesis and provides a potential therapeutic target in hepatocellular carcinoma
Source: Mol Oncol. 2018 Oct 26;12(12):2042–54. doi: 10.1002/1878-0261.12358 (PMC6275258; doi:10.1002/1878-0261.12358)
Supplement: Supplementary file 1 — Fig. S1. Photographs of two TMAs. Fig. S2. Establishment of CHO clones stably expressing integrin αvβ3. Fig. S3. Nogo‐B is remarkably upregulated in different cancers. Fig. S4. Representative photographs of immunohistochemical Nogo‐B staining in normal liver tissue and HCC. [file MOL2-12-2042-s001.pdf]

Supplementary Figures and Figure Legends

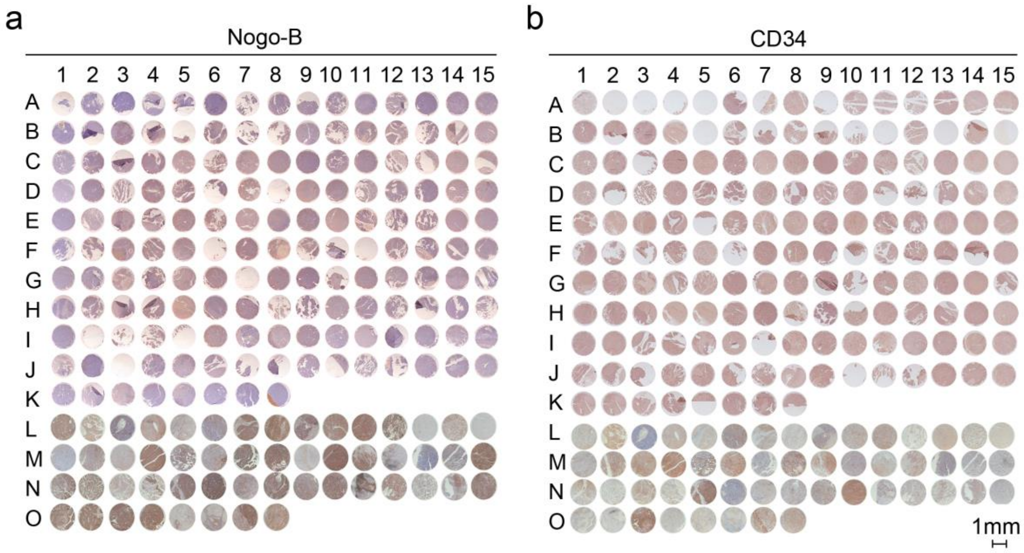

Supplementary Figure 1 Photographs of two TMAs.

TMAs are utilized in expression analysis of Nogo-B (a) and CD34 (b) in human HCC through immunohistochemical staining. Scale bar, 1 mm.

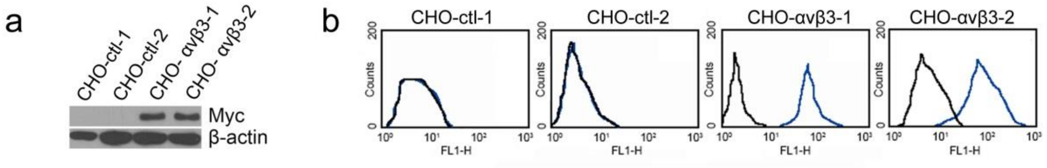

Supplementary Figure 2 Establishment of CHO clones stably expressing integrin αvβ3.

(a) Characterization of CHO cell clones stably expressing integrin $\alpha_v\beta_3$ or vector control transfectants through Western blot using anti-Myc antibody. β-actin serves as a loading control.

(b) Characterization of CHO cell clones through immunofluorescence cytometry using anti-integrin αvβ3 antibody. Black line, control IgG staining. Blue line, LM609 antibody staining.

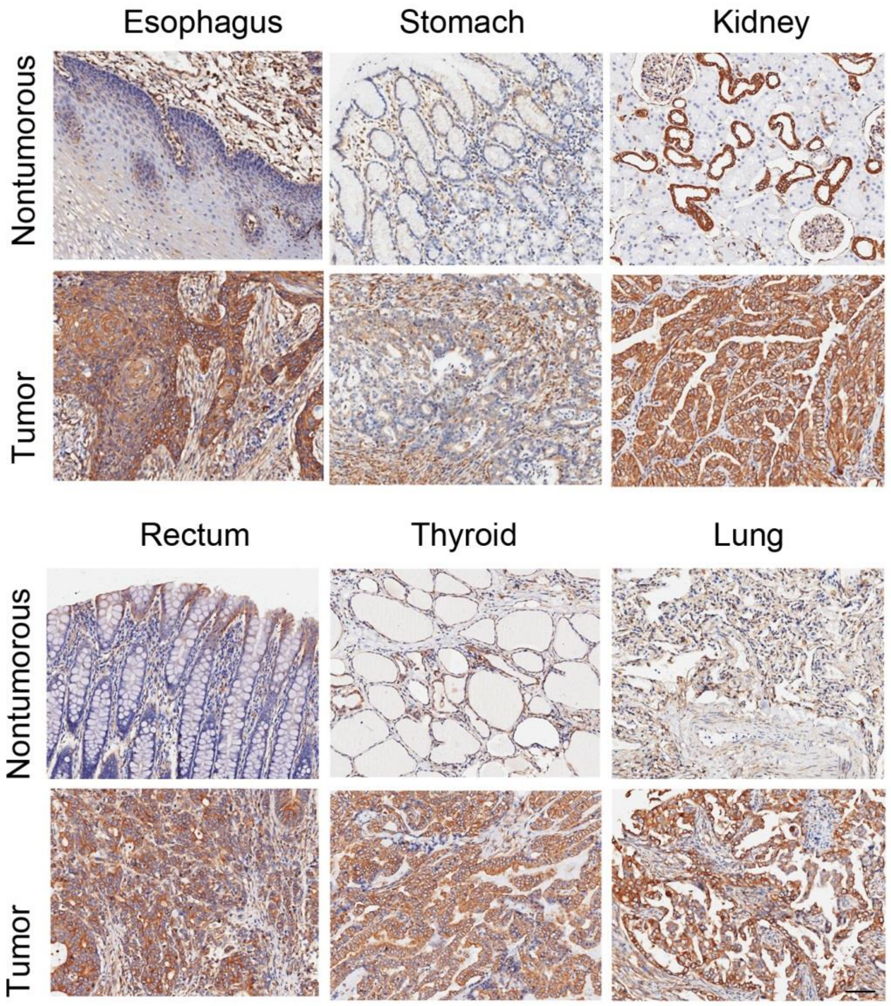

Supplementary Figure 3 Nogo-B is remarkably up-regulated in different cancers. Representative photographs of immunohistochemical Nogo-B staining in six other cancers derived from the indicated tissues. Scale bar, 50 μm.

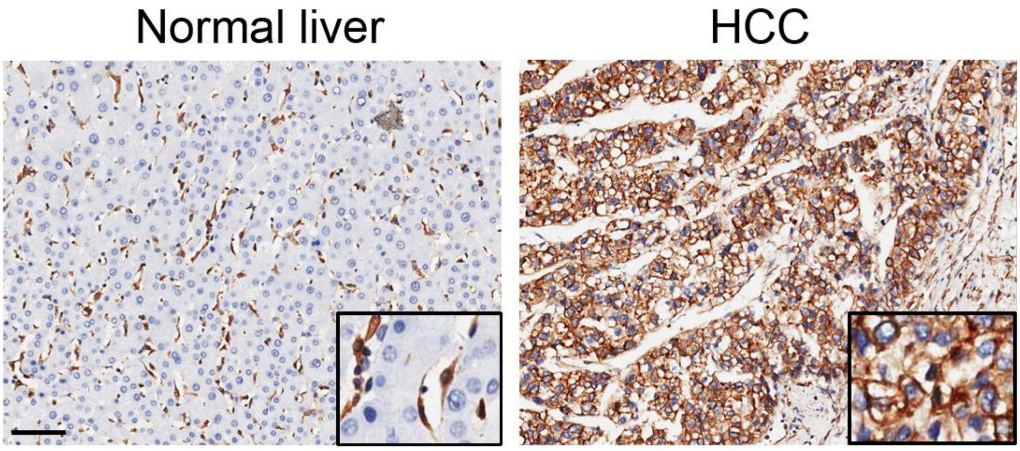

Supplementary Figure 4 Representative photographs of immunohistochemical Nogo-B staining in normal liver tissue and HCC. Scale bar, 50 μm.
